# Optimization of transplantation methods using isolated mesenchymal stem/stromal cells: clinical trials of inflammatory bowel diseases as an example

**DOI:** 10.1186/s41232-024-00350-5

**Published:** 2024-08-16

**Authors:** Daisuke Hisamatsu, Akimi Ikeba, Taku Yamato, Yo Mabuchi, Mamoru Watanabe, Chihiro Akazawa

**Affiliations:** 1 Intractable Disease Research Center, Juntendo University Graduate School of Medicine, 2-1-1 Hongo, Bunkyo-Ku, Tokyo, 113-8421 Japan; 2 Department of Clinical Regenerative Medicine, Fujita Medical Innovation Center, Fujita Health University, Tokyo, Japan; 3 Faculty of Medicine, Juntendo University, 2-1-1 Hongo, Bunkyo-Ku, Tokyo, 113-8421 Japan

**Keywords:** Cell therapy, Clinical trial, Inflammatory bowel disease, Mesenchymal stem/stromal cells, Prospective isolation, Three-dimensional culture

## Abstract

Mesenchymal stem/stromal cells (MSCs) are distributed in various tissues and are used in clinical applications as a source of transplanted cells because of their easy harvestability. Although MSCs express numerous cell-surface antigens, single-cell analyses have revealed a highly heterogeneous cell population depending on the original tissue and donor conditions, including age and interindividual differences. This heterogeneity leads to differences in their functions, such as multipotency and immunomodulatory effects, making it challenging to effectively treat targeted diseases. The therapeutic efficacy of MSCs is controversial and depends on the implantation site. Thus, there is no established recipe for the transplantation of MSCs (including the type of disease, type of origin, method of cell culture, form of transplanted cells, and site of delivery). Our recent preclinical study identified appropriate MSCs and their suitable transplantation routes in a mouse model of inflammatory bowel disease (IBD). Three-dimensional (3D) cultures of MSCs have been demonstrated to enhance their properties and sustain engraftment at the lesion site. In this note, we explore the methods of MSC transplantation for treating IBDs, especially Crohn’s disease, from clinical trials published over the past decade. Given the functional changes in MSCs in 3D culture, we also investigate the clinical trials using 3D constructs of MSCs and explore suitable diseases that might benefit from this approach. Furthermore, we discuss the advantages of the prospective isolation of MSCs in terms of interindividual variability. This note highlights the need to define the method of MSC transplantation, including interindividual variability, the culture period, and the transplantation route.

## Background

The number of patients with inflammatory bowel diseases (IBDs), including ulcerative colitis (UC) and Crohn’s disease (CD), was approximately 5 million worldwide in 2019 [1]. The number is increasing in industrialized countries, including Asia, and is estimated to reach 10 million by 2050 [2, 3]. Although IBDs are caused by chronic inflammation, UC involves inflammation of the rectum to the small intestine, whereas CD involves inflammation of the whole gastrointestinal tract from the gut to the mouth [4]. Inflammation is promoted by genetic and environmental factors such as the gut microbiome [5, 6]. Considering its ability to suppress inflammation, 5-aminosalicylic acid (5-ASA) is prescribed to patients with IBD as a first-line treatment [7]. However, the anti-inflammatory effect of 5-ASA is insufficient for durable remission and mucosal healing [8, 9]. Mucosal healing, denoting the regeneration of the damaged intestinal mucosa, is essential for long-term clinical remission in patients with IBDs [10]. Recently, novel therapeutics, such as biologics and small molecules, have been developed to support mucosal healing. Anti-tumor necrosis factor (TNF)-α therapies (e.g., infliximab and adalimumab) are typical biologics and are often used in patients unresponsive to 5-ASA and corticosteroid treatments [11, 12]. However, a certain population of patients with CD are either unresponsive or lose their response to biologics [13, 14]. Although 70% of patients with CD display small bowel lesions [15], the poor therapeutic efficacy of biologics in treating small bowel lesions might explain the lack of improvement in outcomes, including hospitalization [10, 16, 17]. Another potential reason could be the low treatment efficacy of refractory perianal fistulas [18]. There is no definitive evidence that new biologics and small molecules in long-term use have totally closed intestinal or anal fistula lesions in CD that cause a poor outcome and operation. Therefore, developing novel therapies for long-term clinical remission of patients with CD would be important.

Recently, stem cell transplantation therapy for IBDs has attracted attention because of its immunomodulatory function and promotion of mucosal healing [19, 20]. Mesenchymal stem/stromal cells (MSCs) are good cell source candidates owing to their immunosuppressive effects and ability to migrate to inflammatory sites [20–23]. In the USA, EU, and Japan, darvadstrocel, a dispersion of expanded allogeneic, human adipose tissue–derived MSCs (AD-MSCs), is approved for the treatment of patients with an inadequate response to at least one conventional therapy or biologics with complex perianal fistulas with non-active or mildly active luminal CD [24]. However, a recent announcement underpinned that the primary endpoint of combined remission at 24 weeks could not be achieved in the phase III ADMIRE-CD II study [25]. In addition, a suitable transplantation method for MSCs, including the delivery route (i.e., intravenous, intraperitoneal, or anal injection) and cell form, has not yet been established for the treatment of IBDs [21]. Therefore, further developing stem cell–based therapies and investigating transplantation methods suitable for difficult-to-treat conditions, such as complex perianal fistulas, are necessary.

MSCs are a cell population that adheres to a plastic dish under culture conditions and can be obtained from diverse tissues, such as the bone marrow, adipose tissue, placenta, dental pulp, and skin [26–30]. Although bone marrow–derived MSCs (BM-MSCs) have been the most studied and are used in many clinical applications [31], they are present in only 0.001–0.01% of the bone marrow and exhibit invasive issues at the harvesting stage [32, 33]. Adipose tissues contain the MSC fraction with 1–10% stromal cells and are attracting attention as a cell source for clinical applications owing to their accessibility [34, 35]. Previously, we compared the percentage of the MSC fraction among subcutaneous, amnion, chorion, villus, umbilical cord, and visceral fat and demonstrated that it is the highest in subcutaneous fat [28]. MSCs express specific cell-surface antigens (CD44, CD73, CD90, and CD105) and lack hematopoietic cell antigens (CD14, CD19, CD34, and CD45), endothelial marker (CD31), costimulatory molecules (CD40, CD80, and CD86), and MHC molecules (HLA class II) [26]. The positive markers depend on the species and tissue of origin [27, 36–41]. Considering the differences in cell-surface markers and gene expression profiles [42], the function of MSCs in migration at the inflammation site and their immunomodulatory features may vary [43, 44]. Additionally, it has been noted that the characterization of MSCs is altered during the clinical-scale expansion because of cellular senescence [45–47].

In this paper, we review clinical trials of MSCs in patients with IBDs, especially CD, from 2012 to 2023 in terms of the cell culture period for transplantation, transplantation methods, and their mode of action. Furthermore, we describe our novel findings demonstrating the advantages of isolating MSCs using fluorescence-activated cell sorting (FACS) in mouse and human studies.

## Methods

### Search strategy and study selection

Published papers were sorted using the following bibliographic databases: PubMed (from January 2012 to December 2023) and Google Scholar (from January 2012 to December 2023). The search terms in Table 1 include variations of “inflammatory bowel disease,” “Crohn’s disease,” “ulcerative colitis,” and “mesenchymal stem/stromal cells.” The search terms related to Table 2 included variations of “three-dimensional,” “organoid,” “spheroid,” “scaffold,” “spheroid-free,” “tissue engineering,” and “mesenchymal stem/stromal cells.”

**Table 1:** Published clinical trials related to CD

No	Auto/Allo	Tissue of origin	No. of cells	Culture period	Delivery route	No. of patients	Efficacy	Proposed mode of action	Type of CD	Phase	Ref
1	Auto	BM	2–10 × 10^6^/kg	14 ± 4 days	i.v.	16	45% (5/11) clinical response (2 weeks)	Resetting immune functionalities	Luminal	Phase 1	Dhere et al., 2016 [48]
2	Allo	BM	3 × 10^6^/kg	UNK	i.v.	50	70% (21/30) complete clinical remission (2 months)	Inhibiting the inflammatory process and stimulating tissue regeneration by reducing proinflammatory cytokine productions at the inflamed site	Luminal	UNK	Knyazev et al., 2013 [49]
3	Allo	BM	2 × 10^6^/kg	UNK (less than P5)	i.v.	16	80% (12/15) clinical response and 53% (8/15) clinical remission (42 days)	Immunopathogenic mechanism	Luminal	Phase 2	Forbes et al., 2014 [50]
4	Allo	BM	1.5–2.0 × 10^6^/kg	4 weeks	i.v.	13	15% (2/13) clinical response (8 weeks), one of whom achieved clinical remission	Increasing NK cell proliferation, leading to MSC lysis, and/or decreasing the intestinal infiltration of NK/NKT cells	Luminal	Phase 1–2	Gregoire et al., 2018 [51]
5	Allo	UC	1 × 10^6^/kg	UNK	i.v.	82	0% (0/41) complete remission The CDAI, HBI, and CS dosage significantly decreased in the treated group compared with those in the control group	MSC therapy has moderate immunomodulatory effects	Luminal	Phase 1–2	Zhang et al., 2018 [52]
6	Allo	BM	1.5–2.0 × 10^8^ (i.v.), 8 × 10^7^ (i.l.)	UNK	i.v., i.l.	36	67% (8/12) simple fistula healing (3 and 6 months)	Regulating immune response and reducing inflammation	Perianal fistulas	Phase 2	Knyazev et al., 2020 [53]
7	Allo	BM	1–9 × 10^7^	Over 2 weeks (maximally P3)	i.l.	21	53% (8/15) fistula healing (6 weeks) Low-dose (1–3 × 10^7^ cells) administration promoted perianal fistula healing	Immunosuppressive effects at the local site	Perianal fistulas	Phase 1–2	Molendijk et al., 2015 [54]
8	Allo (Remestemcel-L)	BM	1.5–3.0 × 10^8^	UNK	i.l.	6	0% (0/4) clinical remission The simple endoscopic scores decreased by 71% (treated group) and increased by 61% (control group) (3 months)	No description	Luminal	Phase 1–2	Lightner et al., 2022 [55]
9	Allo	BM	7.5 × 10^7^	UNK (less than P5)	i.l.	22	31% (4/13) (treated group) and 20% (1/6) (control group) complete clinical and radiographic healing (6 months)	No description	Peripouch fistulas	Phase 1–2	Lightner et al., 2023 [56]
10	Allo	BM	3 × 10^7^	4 weeks (P3)	i.l.	10	40% (4/10) complete resolution of the stricture (48 weeks)	Anti-inflammatory properties and anti-fibrotic effects	Luminal stricture	Phase 1–2	Vieujean et al., 2022 [57]
11	Allo	BM	7.5 × 10^7^	UNK (less than P5)	i.l.	23	83% (15/18) (treated group) and 40% (2/5) (control group) complete clinical and radiographic healing (6 months)	No description	Complex perianal fistulas	Phase 1–2	Lightner et al., 2023 [58]
12	Allo	AT	2–4 × 10^7^	UNK	i.l.	24	69% (9/13) reduction in draining fistulas, and 56% (9/16) complete closure of the treated fistula (24 weeks)	Reducing chronic inflammation in the fistula by immunomodulatory signals (anti-inflammation) and releasing trophic factors with regenerative properties (anti-fibrosis)	Complex perianal fistulas	Phase 1–2	Portilla et al., 2013 [59]
13	Allo (Darvadstrocel)	AT	1.2 × 10^8^	UNK	i.l.	212	50% (53/107) (treated group) and 34% (36/105) (placebo group) combined remission (24 weeks)	Inducing IDO and subsequent degradation of tryptophan to kynurenine in the presence of inflammatory mediators (immunomodulatory effects)	Complex perianal fistulas	Phase 3	Panés et at., 2016 [60]
14	Allo (Darvadstrocel)	AT	1.2 × 10^8^	UNK	i.l.	22	59% (13/22) and 68% (15/22) combined remission (24 and 52 weeks, respectively)	Inhibiting T cell function, increasing Treg, reducing proinflammatory cytokine production, and increasing anti-inflammatory cytokine production (i.e., immunomodulatory effects)	Complex perianal fistulas	Phase 3	Furukawa et al., 2023 [61]
15	Allo (TH-SC01)	UC	1.2 × 10^8^	15 days before first passage (cells cultured to P5)	i.l.	10	60% (6/10) combined remission, and 70% (7/10) clinical response (24 weeks)	Immunomodulatory functions	Complex perianal fistulas	Phase 1	Wei et al., 2023 [62]
16	Auto	AT	2 × 10^7^	UNK (3–6 days for culture in a bioreactor with matrix)	i.l. (with scaffold)	12	83% (10/12) complete clinical healing and radiographic markers of response (6 months)	No description	Perianal fistulas	Phase 1	Dietz et al., 2017 [63]
17	Auto	AT	3.5 × 10^7^	UNK (placement of the MSC-coated plug after 6 weeks)	i.l. (with scaffold)	5	60% (3/5) complete cessation of drainage, and 40% (2/5) reduction in drainage (6 months)	No description in the function of MSCs. An implantable matrix is used for sustained local exposure throughout the fistula tract	Rectovaginal fistulas	Phase 1	Lightner et al., 2020 [64]
18	Auto	AT	2 × 10^7^	UNK (placement of the MSC-loaded plug after 6 weeks)	i.l. (with scaffold)	20	78% (14/18) complete clinical healing and 67% (12/18) MRI response (6 months)	Immunomodulatory functions of MSCs. An implantable matrix is used for sustained local exposure throughout the fistula tract	Complex perianal fistulas	Phase 1	Dozois et al., 2023 [65]

*Allo* allogeneic, *AT* adipose tissue, *Auto* autologous, *BM* bone marrow, *CS* corticosteroid, *CD* Crohn’s disease, *CDAI* Crohn’s disease activity index score, *HBI* Harvey-Bradshaw index, *IDO* indoleamine 2,3-dioxygenase, *i.a.* intraarterial, *i.l.* intralesional, *i.v.* intravenous, *MRI* magnetic resonance image scan, *MSC* mesenchymal stem/stromal cell, *NK* natural killer, *P* passage, *Treg* regulatory T cells, *UC* ulcerative colitis, *UC* umbilical cord, *UNK* unknown

**Table 2:** Published clinical trials using 3D MSC constructs

No	Diseases	Auto/Allo	Tissue of origin	No. of cells	3D culture/scaffold	Culture period	No. of patients	Efficacy	Proposed mode of action	Phase	Ref
1	Degenerative disc disease and isthmic spondylolisthesis	Auto	AT	5.3 × 10^6^	Yes/free	50 ± 6 days (less than P5) for expansion 40 ± 14 days for osteogenic differentiation and 3D culture	3	Improved the mean VAS score and the ODI (12 months)	Promoting angiogenesis and osteogenesis leads to bone reconstitution	Clinical proof of concept	Fomekong et al., 2017 [66]
2	Knee chondral lesions	Auto	Synovium	6 × 10^7^	Yes/free	UNK (less than P2 for expansion) 2–3 weeks for treatment with ascorbate and 3D culture	5	Improved all clinical scores (pain, symptoms, activities of daily living, sports activity, and QOL) (24 months)	Unknown, whether directly (differentiation to chondrocytes) or indirectly (releasing mediators that enhanced repair by endogenous cartilage cells)	Case series; Level of evidence, 4	Shimomura et al., 2018 [67]
3	Atrophic pseudarthrosis	Auto	BM	0.5–2.0 × 10^6^/clot	No/auto fibrin clot	10–18 days (P1) for expansion 4 days for osteogenic differentiation	8	100% (8/8) positive clinical outcomes with recovering limb function (6.7 months)	Regenerative (self-renewal and differentiation capacities) and trophic properties	UNK	Giannotti et al., 2013 [68]
4	Knee cartilage lesions	Auto	AT	3.9 × 10^6^	UNK/fibrin glue	UNK	54	The mean IKDC score and the TAS improved in the MSC with scaffold- and the MSC without scaffold-treated groups (28.6 months). No significant differences between the two groups	Differentiation ability to chondrocytes and paracrine effects of secreted factors. The fibrin glue induces better cell survival, proliferation, differentiation, and matrix synthesis	Cohort study; Level of evidence, 3	Kim et al., 2015 [69]
5	Knee chondral defects	Auto	Synovium	8 × 10^6^	Yes/type I/III Col membrane (Chondro-Gide)	UNK (less than P3 for expansion) 2 days for culture with the scaffold	14	The clinical outcome improved in the MSC-treated and the chondrocyte-treated groups (24 months). The MSC-treated group scored significantly better than the chondrocyte-treated group	Trophic, immunoregulatory, and anti-inflammatory effects. The SD-MSCs have greater chondrogenic and less osteogenic potential than BM-MSCs	Randomized study	Akgun et al., 2015 [70]
6	Periodontal intrabony defects	Auto	BM	3.5 × 10^7^	No/woven-fabric composite scaffold and PRP	1 month	10	Improved all three parameters (CAL, VPD, and linear bone growth)	Differentiating into periodontal cells. The signaling molecules contained in PRP may stimulate cell migration, proliferation, and differentiation	Phase 1–2	Baba et al., 2016 [71]
7	Bone defects	Auto	BM	1.2 × 10^4^	No/β-TCP	No culture period	41	100% (41/41) complete healing with a mean time of 5.04 ± 2.11 months	No description in the function of MSCs. BM-MSCs can rapidly adhere to the β-TCP, resulting in the efficient construction of an MSC/β-TCP composite	Prospective study	Zhuang et al., 2017 [72]
8	Femoral bone defects	Auto	BM	15 ± 4.5 × 10^6^	UNK/β-TCP	4 weeks (P4)	37	The mean Harris hip score improved in the MSC with the scaffold-treated, scaffold-treated, and control groups (20.7, 32.0, and 17.3 months, respectively). No significant differences among them	Secretion of various growth factors and cytokines	Phase 2a	Sponer et al., 2018 [73]
9	Maxillary cystic bone defects	Auto	Alveolar BM	5–10 × 10^6^	Yes/GA-cross-linked 3D serum matrix	UNK (P3) 20–30 days for osteogenic differentiation with the scaffold	9	The mean rate of the CT values in the treated area was 2.52, and that of the control area of spongy alveolar bone was 0.99 (7 months)	No description in the function of MSCs. The autologous serum scaffold avoids immunogenic effects and can stimulate vascularization, cell growth, and osteogenesis	Phase 1–2	Redondo et al., 2018 [74]
10	Severe mandibular ridge resorption	Auto	BM	20 × 10^6^/cm^3^	No/BCP	Over 3 weeks (P1)	13	100% (11/11) significant increase in the total bone volume (12 months)	Inducing wound healing by paracrine effects	Phase 1	Gjerde et al., 2018 [75]
11	Full-thickness rotator cuff tears	Auto	BM	20 × 10^6^	No/type I Col membrane (OrthADAPT™)	2 weeks	13	38% (3/8) (treated group) and 40% (2/5) (control group) healing rotator cuff (12 months). 38% (3/8) (treated group) adverse events (e.g., the recurrence of rupture). The study was withdrawn	No description in the function of MSCs. MSCs alone are insufficient to improve the healing of repaired tendons, necessitating scaffolds for augmentation	A Level 1 evidence treatment study	Lamas et al., 2019 [76]
12	Periodontal intrabony defects	Auto	Gingiva	0.2–8.0 × 10^6^/cm^3^	Yes/β-TCP	Over 2 weeks (P2) 2 days for culture with the scaffold	20	Clinical outcomes (VPD and CAL gain) significantly decreased in the treated group compared with the control group (6 months)	Changing the components of the gingival fibroblasts’ ECM at the periodontal defect site	Randomized clinical trial	Abdal-Wahab et al., 2020 [77]
13	Periodontitis	Auto	Alveolar BM	5 × 10^6^	No/Col enriched with auto fibrin/platelet lysate	18–24 days (P2)	27	100% (9/9) significant clinical improvements in the MSC-treated and Col-treated groups (12 months). No significant differences between the two groups	Paracrine effects stimulating crosstalk with host cells and cell recruitment. Disease microenvironments (periodontal pockets) may significantly affect the efficacy of MSCs	Phase 1–2	Apatzidou et al., 2021 [78]
14	Osteoarthritis	Allo	UCB	1.2–2.0 × 10^7^	UNK/HA hydrogel	Over 2 weeks	7	Improved the mean VAS score and IKDC score (24 weeks)	Paracrine effects promoting the differentiation of endogenous chondroprogenitors	Phase 1–2	Park et al., 2017 [79]
15	Cranial defects	Allo	BM	2.5 × 10^6^/ml	Yes/β-TCP (ChronOS granules)	UNK (less than P4 for expansion) 2 days for culture with the scaffold	10	100% (3/3) good restoration of the cranial contour (3 and 6 months). 100% (3/3) resorption of the construct (12 months). The trial is ongoing	Differentiation capacities and secretory properties that promote tissue regeneration. MSCs alone cannot restore bone structure and require a supportive matrix	Phase 1	Morrison et al., 2018 [80]
16	Acute complete spinal cord injury	Allo	UC	4 × 10^7^	No/Col (NeuroRegen) scaffold derived from decellularized bovine aponeurosis	UNK	2	100% (2/2) improvement of the ASIA impairment scale (from A to C) (12 months)	Immunomodulatory and neurotrophic effects on spinal cord injury repair. The scaffold functionalized with MSCs might induce neuronal differentiation of endogenous NSCs to improve functional recovery	Phase 1	Xiao et al., 2018 [81]
17	Degenerative disc disease and spondylolisthesis	Auto	BM	0.3–1.0 × 10^6^	Yes/Allo cancellous bone graft	21 days 4 h for culture with cancellous bone cubes	73	The posterior spinal fusion rate and the complete treatment response were significantly higher in the MSC-treated group (76% and 71%, respectively) than in the control group (51% and 51%, respectively) (12 months)	Higher osteogenesis than the gold-standard graft material	Phase 1–2	Garcia de Frutos et al., 2020 [82]
18	Chronic skin ulcers	Allo	Wharton’s jelly	2 × 10^6^	UNK/Acellular amniotic membrane	UNK	5	The wound healing time and wound size significantly decreased (6 and 9 days)	Improved dermal matrix deposition via secreted factors. The amniotic membrane has benefits (antiadhesive and bacteriostatic effects, wound protection, pain reduction, and epithelialization enhancement)	Randomized clinical trial	Hashemi et al., 2019 [83]
19	Chronic ischemic heart disease	Allo	UC	1 × 10^8^	UNK/Bovine Col	UNK (P5)	50	The mean infarct size percentage change (− 3.1%, 5.2%, and 8.6%) in the MSC-plus-collagen treated, the MSC-treated, and the control groups, respectively (12 months)	Generating new myocardial tissue or releasing molecules that harness endogenous repair	Phase 1	He et al., 2020 [84]

*Allo* allogeneic, *AT* adipose tissue, *Auto* autologous, *ASIA* American spinal injury association, *BCP* biphasic calcium phosphate, *BM* bone marrow, *β-TCP* β-tricalcium phosphate, *CAL* clinical attachment loss, *Col* collagen, *CT* computerized tomography, *ECM* extracellular matrix, *GA* glutaraldehyde, *HA* hyaluronic acid, *IKDC* International Knee Documentation Committee, *MSC* mesenchymal stem/stromal cell, *NSCs* neural stem cells, *ODI* Oswestry disability index, *P* passage, *PRP* platelet-rich plasma, *QOL* quality of life, *SD-MSCs* synovium-derived MSCs, *TAS* Tegner activity scale, *3D* three-dimensional, *UC* umbilical cord, *UCB* umbilical cord blood, *UNK* unknown, *VPD* vertical pocket depth, *VAS* visual analog scales

Clinical trials were extracted from the papers searched by one author. Another author validated the list and selected reports that matched the purpose of this review. Eighteen papers of 29 cases were selected in Table 1. Complex perianal fistulas not associated with IBDs were excluded. The cases using adipose-derived stromal vascular fraction were excluded. Nineteen papers of 20 cases were selected in Table 2. MSC preparations consisting of cell sheets were excluded.

### mRNA sequencing (mRNA-seq) and data reanalysis

Principal component and gene expression analyses of MSC markers were performed based on an mRNA-seq dataset from our previous study [85]. The Euclidian distance was calculated based on the total genes (81,602 transcripts) after log2 transformation in the mRNA-seq dataset. Data analyses and visualizations were conducted using the RStudio environment and a Tag-Count Comparison Graphical User Interface (TCC-GUI).

### Statistical analysis

All statistical analyses were performed using the statistical programming language R version 4.3.3 (2024–02-29). Statistical significance was determined using the Wilcoxon rank-sum test.

## Main

### Current status of clinical applications of MSCs in IBD treatment from published papers

Eighteen clinical trials for treating CD have been published in the past decade (Table 1) [48–65]. Of the 18 studies, 10 used BM-MSCs, six used AD-MSCs, and two used umbilical cord–derived MSCs (UC-MSCs) as transplanted cells. These trials included four cases using MSC preparations (i.e., remestemcel-L, darvadstrocel, and TH-SC01) [55, 60–62]. Five of the 18 studies were trials on the intravenous injection of MSCs in patients with luminal CD [48–52]. One study used autologous BM-MSCs for intravenous injection, whereas others used allogeneic BM- or UC-MSCs. No such trials have been conducted using AD-MSCs. For the treatment of fistulizing CD, infusion into the lesion site was performed because of accessibility, with 13 of the 18 reports involving intralesional injections [54–62]. Of the studies using intralesional injections, nine targeted perianal fistulas, two targeted peripouch or rectovaginal fistulas, and two targeted luminal CD, including strictures. One of these reports showed the serial intravenous and intralesional injections of allogeneic BM-MSCs in patients with perianal fistulizing CD [53]. Three studies reported the efficacy and safety of combination therapy with autologous AD-MSCs and bioabsorbable matrices as a scaffold in intralesional injections [63–65]. All the studies used allogeneic MSCs in intralesional injections without scaffolds. Although some trials have reported a few adverse effects, it is important to note that all studies unequivocally demonstrated the safety of MSC transplantation. Almost all studies have shown benefits regarding the efficacy of MSC therapy; however, in a few cases (i.e., patients with luminal CD), they have not shown a significant advantage. For example, Lightner et al. performed intralesional transplantation of allogeneic BM-MSCs (1.5–3.0 × 10^8^) into four patients with luminal CD [55]. They reported that the indices of clinical remission and response, with a simple endoscopic score for CD (SES-CD), dropped from 17 to 5, the Crohn’s disease activity index score (CDAI) dropped from 228 to 200, and C-reactive protein (CRP) remained at 0.20–0.30. In contrast, in the control group, the SES-CD increased from 15.5 to 25.0, the CDAI increased from 146 to 158, and the CRP dropped from 3.65 to 1.50 at 3 months. No patients in the treatment group showed clinical remission or response. One patient received anti-TNF-α monoclonal antibody treatment (i.e., Cimzia) before MSC therapy. Previous studies have shown that patients with pre-treated anti-TNF-α treatment have a lower response to subsequent therapies than naive patients [86, 87]. Lightner et al. indicated that achieving efficacy when attempting new therapeutics for treatment-refractory patients is challenging. They also performed intralesional transplantation of allogeneic BM-MSCs (7.5 × 10^7^) to treat peripouch fistulas in patients with ileal pouch-anal anastomosis [56]. Four of 13 patients (31%) in the treated group and one of six (20%) in the control had complete clinical and radiographic healing at 6 months. Vieujean et al. showed a 40% complete stricture resolution at 48 weeks in patients with luminal CD [57]. On the other hand, Molendijk et al. demonstrated the efficacy of intravenous injection of allogeneic BM-MSCs in treating refractory perianal fistulas in patients with CD [54]. Low-dose administration (3 × 10^7^) resulted in greater fistula healing than high-dose administration (9 × 10^7^). The dose in Lightner’s trial was higher than that in Molendijk’s trial; this may also have caused these differences in efficacy. Thus, further analysis is needed to determine the relationship between efficacy and dose dependency on the CD condition, especially luminal CD.

The culture period of MSCs for transplantation is yet to be clearly defined. Of the 18 reports, only three clearly described the total culture period [48, 51, 57]. Others were either partially or not described. In the papers described, the shortest culture period was 2 weeks, and the maximum was 4 or 6 weeks in five or fewer passages. Previous studies have shown that MSCs gradually lose specific capacities such as proliferation, differentiation, and secretion during expansion [45–47]. Moreover, there are differences in transcriptional characteristics between cultured and freshly isolated MSCs [42]. Even within the same MSC preparation, functions may differ depending on the culture period. Thus, culture days and the number of passages before transplantation should be defined for optimal efficacy.

The key players in the pathogenesis of IBDs are T helper (Th) cells and regulatory T cells (Tregs) [88, 89]. The imbalance in these cells activates other immune cells (i.e., macrophages and B cells) exacerbating inflammation in the gut mucosa [5]. Almost all studies have proposed an immunomodulatory function as a mode of action for MSC therapy. In a preclinical study, MSCs inhibited the function of Th cells and increased the population of Tregs by modulating the secretion of numerous factors, such as pro-inflammatory (e.g., interferon-γ, TNF-α, and interleukin [IL]-17) and anti-inflammatory (IL-10 and transforming growth factor-β) cytokines [90, 91]. Despite analyses of lymphocytes in peripheral blood and biopsy samples, the mode of action of MSCs in humans still needs to be fully understood.

### Clinical trials using the three-dimensional construct of MSCs

Previous studies have shown that spheroids derived from three-dimensional (3D) MSC cultures exhibit enhanced functions such as immunomodulatory effects, angiogenesis, and multipotency by altering the expression of genes such as TNF-α-stimulated gene 6 (TSG-6), fibroblast growth factor-2 (FGF-2), angiogenin, and OCT4 [92–94]. Preclinical studies demonstrated the advantage of 3D culture–derived MSC spheroid transplantation in several animal models [95–99]. In the model of skin ulcers and bone defects, as surgically accessible tissues, the intralesional injection of MSC spheroids led to markedly superior and faster regeneration than that seen with 2D-cultured MSCs [95–97]. These results suggest that these phenotypes are attributed to enhanced gene expression related to the extracellular matrix (ECM), angiogenesis, and migration (e.g., fibronectin, FGF-1, CXC chemokine receptor 4, and integrin α2). In myocardial infarction models, functional recovery was higher in the case of the intramyocardial injection of MSC spheroids than that of 2D culture–derived MSCs [98]. Xu et al. demonstrated that the intralesional injection of AD-MSC spheroids was more beneficial to functional recovery than that of 2D culture–derived MSCs in a kidney injury model [99]. In clinical trials of IBD treatment, the scaffold has been used to sustain transplanted MSCs at the lesion site for an extended time. However, no clinical trials have been conducted on treating IBDs using 3D-structured MSCs without scaffolds. Here, we describe the published clinical trials using 3D structures with MSCs for other diseases (Table 2) [66–84].

Seventeen of the 19 studies used bioabsorbable scaffolds, whereas two did not. In scaffold-free trials, 3D structures were generated by expanding cultures of autologous MSCs, followed by 3D cultures in osteogenic differentiation media or media containing ascorbate [66, 67]. 3D cultures have been performed to improve the effectiveness of bone reconstitution by facilitating osteogenesis and angiogenesis or improving adhesiveness to the cartilaginous matrix, which has been used to treat degenerative disc disease or knee chondral lesions. In contrast, in the trials with the MSC plus scaffold construct, 13 papers were trials for osteochondral and tendon regeneration, two for spinal cord and spine regeneration, one for wound healing of ulcers, and one for ischemic heart disease. Fourteen of the 19 papers used 3D constructs for bone and cartilage regeneration, indicating that surgically accessible tissues were targeted [67–80]. MSCs originated from various tissues, including the bone marrow (eight reports), umbilical cord (three reports), adipose tissue (two reports), synovium (two reports), alveolar bone (two reports), gingiva (one report), and Wharton’s jelly (one report). Lamas et al. reported treating patients with full-thickness rotator cuff tears using autologous BM-MSCs with type I collagen membrane [76]. They used a scaffold to augment the ability of MSCs because MSCs alone are insufficient to improve the healing of repaired tendons. Morrison et al. also reported that MSCs alone cannot restore bone structure and that a supportive matrix is required [80]. Depending on the tissue of origin, MSCs have different differentiation abilities [41]. Zhuang et al. used BM-MSCs for treating bone defects because the MSCs can prompt attachment to the surface and inner space of porous scaffolds, such as a β-tricalcium phosphate, efficiently constructing a bioactive composite [72]. Although synovium-derived MSCs have more prominent chondrogenic and less osteogenic differentiation abilities than those derived from the bone marrow or periosteum, Akgun et al. used synovium-derived MSCs with type I/III collagen membranes to treat chondral defects of the knee [70]. Apatzidou et al. reported improvements in osteoarthritis using umbilical cord blood-derived MSCs, which promoted the differentiation of endogenous chondroprogenitors through paracrine effects [78]. In the case of periodontal intrabony defects, autologous gingiva-derived MSCs alter the components of the extracellular matrix in gingival fibroblasts by migrating into periodontal defects [77]. Thus, several trials employ MSCs to promote the multipotency of MSCs or the differentiation of endogenous cells in the lesion site rather than immunomodulatory functions; therefore, the MSCs used tend to differ depending on the type of scaffold used or the target disease.

Regarding the culture period, although eight studies did not describe the MSC expansion time, the period ranged from 14 to 50 days in the papers described. Generating 3D constructs without scaffolds requires a longer culture period (2–3 weeks or 40 days) [66, 67]. In contrast, when using scaffolds, the incubation period was mostly 2 days, with the shortest period being 4 h and the longest being 20–30 days. In particular, 3D cultures with osteogenic differentiation require extended culture periods. Notably, eight of the 19 reports performed in vitro 3D culture, whereas the others reported short incubation times ranging from 10 to 60 min in syringes or lacked details. Because the 3D structure of MSCs by self-aggregation enhances their immunomodulatory effects, a scaffold-free 3D MSC construct for treating inflammatory diseases, including IBDs, may be useful in clinical trials. However, evidence on the efficacy of MSC spheroids in clinical trials is currently insufficient; in the future, it is considered necessary to advance clinical research, including IBDs.

### Advantages of prospective isolated MSCs using specific cell-surface markers

**Fig. 1:**
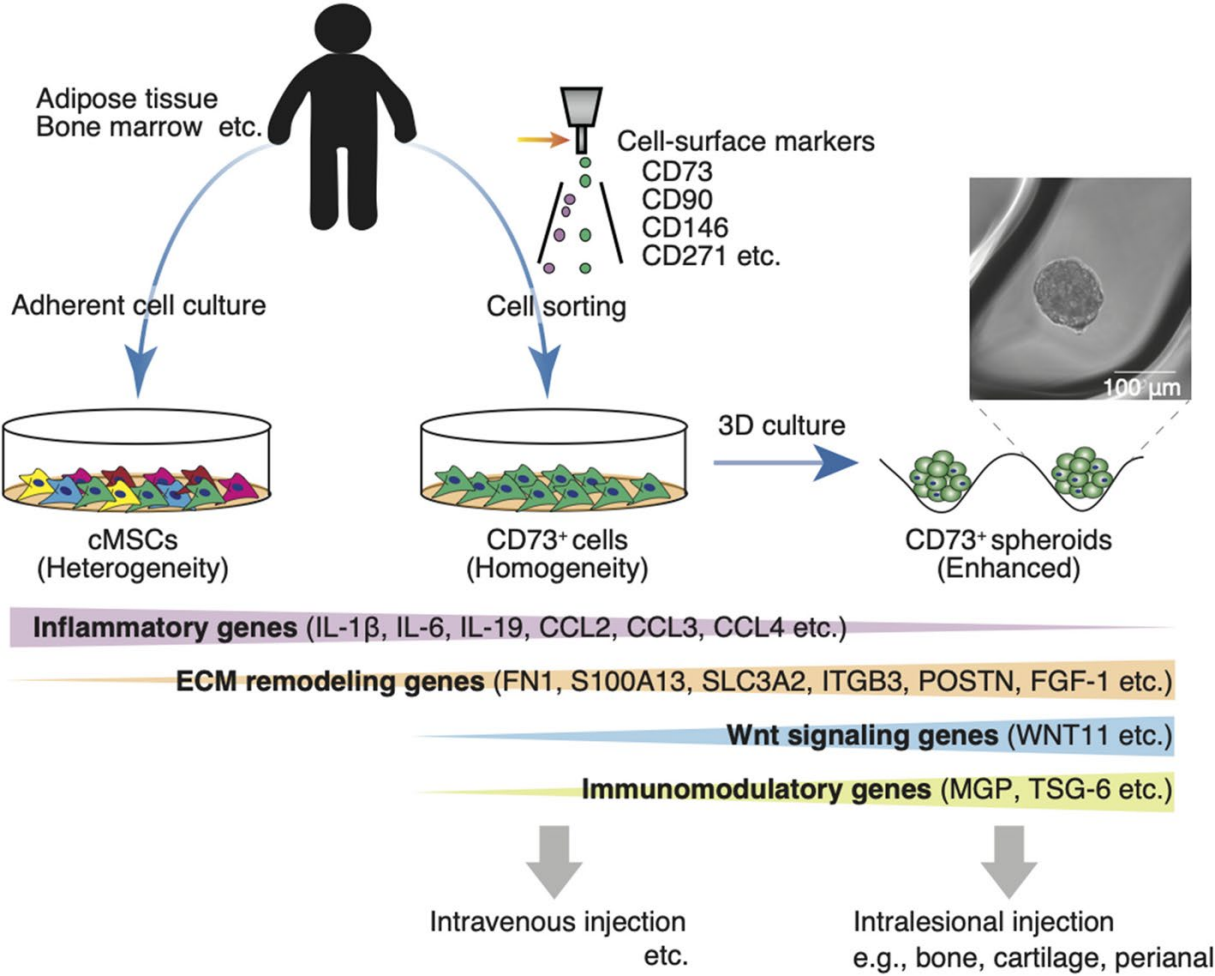
Advantage of prospective isolated CD73^+^ cells and their three-dimensional (3D) culture. Conventional mesenchymal stem/stromal cells (cMSCs) are a heterogeneous cell population that adheres to a plastic dish under culture conditions. CD73^+^ cells are a homogeneous cell population prospectively isolated using a cell sorter. Compared with cMSCs, CD73^+^ cells showed the downregulation of inflammatory gene expressions and the upregulation of extracellular matrix remodeling gene expressions. The 3D culture-derived spheroids of CD73^+^ cells (CD73^+^ spheroids) enhance these gene expression profiles. Additionally, CD73^+^ spheroids have elevated gene expressions related to Wnt signaling and immunomodulation compared with CD73.^+^ cells. CCL, C–C motif chemokine ligand; ECM, extracellular matrix; FN1, fibronectin 1; IL, interleukin; ITGB3, integrin β3; MGP, matrix Gla protein; MSCs, mesenchymal stem/stromal cells; POSTN, periostin; 3D, three-dimensional; TSG-6, tumor necrosis factor-α-stimulated gene 6

MSCs exhibit cellular heterogeneity with various cell-surface antigens [100]. Even when similar cell-surface markers are expressed on MSCs, their functions, including differentiation potential and immunomodulatory effects, differ depending on the tissue of origin. For example, previous studies have shown that UC-MSCs have higher proliferation, colony-forming, and immunomodulation abilities than AD-MSCs [101, 102]. Furthermore, it is noted that adhering MSCs on plastic dishes are heterogeneous cell populations with donor-to-donor variability in clinical trials [58, 103]. This interindividual variability is a significant problem when autologous MSCs are used. In preclinical studies, high-quality BM-MSCs have been isolated using several specific markers by FACS (i.e., for mice, a combination of CD140a and Sca-1; for humans, a combination of CD90, CD271, and CD146) [27, 37]. These prospectively isolated MSCs have a high potential for colony formation. In addition, we previously demonstrated that AD-MSCs isolated using CD73 molecules (termed CD73^+^ cells) have a higher colony-forming ability than conventional heterogeneous adherent MSCs (termed cMSCs) from human and rodent experiments [28]. Intranasal injection of CD73^+^ cells reduces macrophage infiltration and suppresses fibrosis at the lesion site in mice with bleomycin-induced pulmonary fibrosis [28]. Moreover, in a mouse model of dextran sulfate sodium-induced colitis, intravenous injection of CD73^+^ cells significantly attenuated tissue destruction compared to that observed with cMSCs [85]. We also demonstrated that compared with cMSCs, human adipose tissue–derived CD73^+^ cells downregulated the expression of genes related to “immune response,” “inflammatory response,” and “neutrophil chemotaxis” (e.g., IL-1β, IL-6, IL-19, C–C motif chemokine ligand 2 [CCL2], CCL3, and CCL4), as revealed via a transcriptome analysis (Fig. 1) [85]. In contrast, the terms for the upregulated genes were “GTP biosynthetic process,” “UTP biosynthetic process,” “negative regulation of platelet-derived growth factor receptor signaling pathway,” and “negative regulation of intrinsic apoptotic signaling pathway.” These features indicate that CD73 is an ectonucleotidase that metabolizes extracellular adenosine triphosphate [104].

Our recent study also revealed upregulation of the expression of ECM-related genes (e.g., fibronectin 1, S100A13, SLC3A2, integrin β3, periostin, and FGF-1) in CD73^+^ cells compared with that in cMSCs (Fig. 1) [85]. Furthermore, the 3D culture of CD73^+^ cells enhanced their characteristics, such as upregulation of the expression of ECM remodeling and Wnt signaling genes (e.g., WNT11) and downregulation of the expression of inflammatory genes (Fig. 1). Upregulation of the expression of immunomodulatory genes (e.g., matrix Gla protein and TSG-6) was also observed in 3D culture-derived CD73^+^ spheroids (Fig. 1). Considering these properties, CD73^+^ spheroids may be suitable for transanal transplantation to treat IBDs. Indeed, our preclinical study showed that CD73^+^ spheroids increased the engraftment rate into the lesion site compared to CD73^+^ cells, preventing mucosal atrophy in a mouse model of colitis. The proportion of endogenous CD140a^+^ fibroblasts was altered after CD73^+^ spheroid transplantation in the lesion site. Supernatants of CD73^+^ spheroids, including their secretory factors, affect the gene expression profiles of fibroblasts, such as ECM remodeling and integrin downstream genes in vitro. These findings suggest that transanal transplantation of CD73^+^ spheroids exerts a potential therapeutic effect against IBDs via the paracrine effects of secreted factors caused by sustained engraftment.

**Fig. 2:**
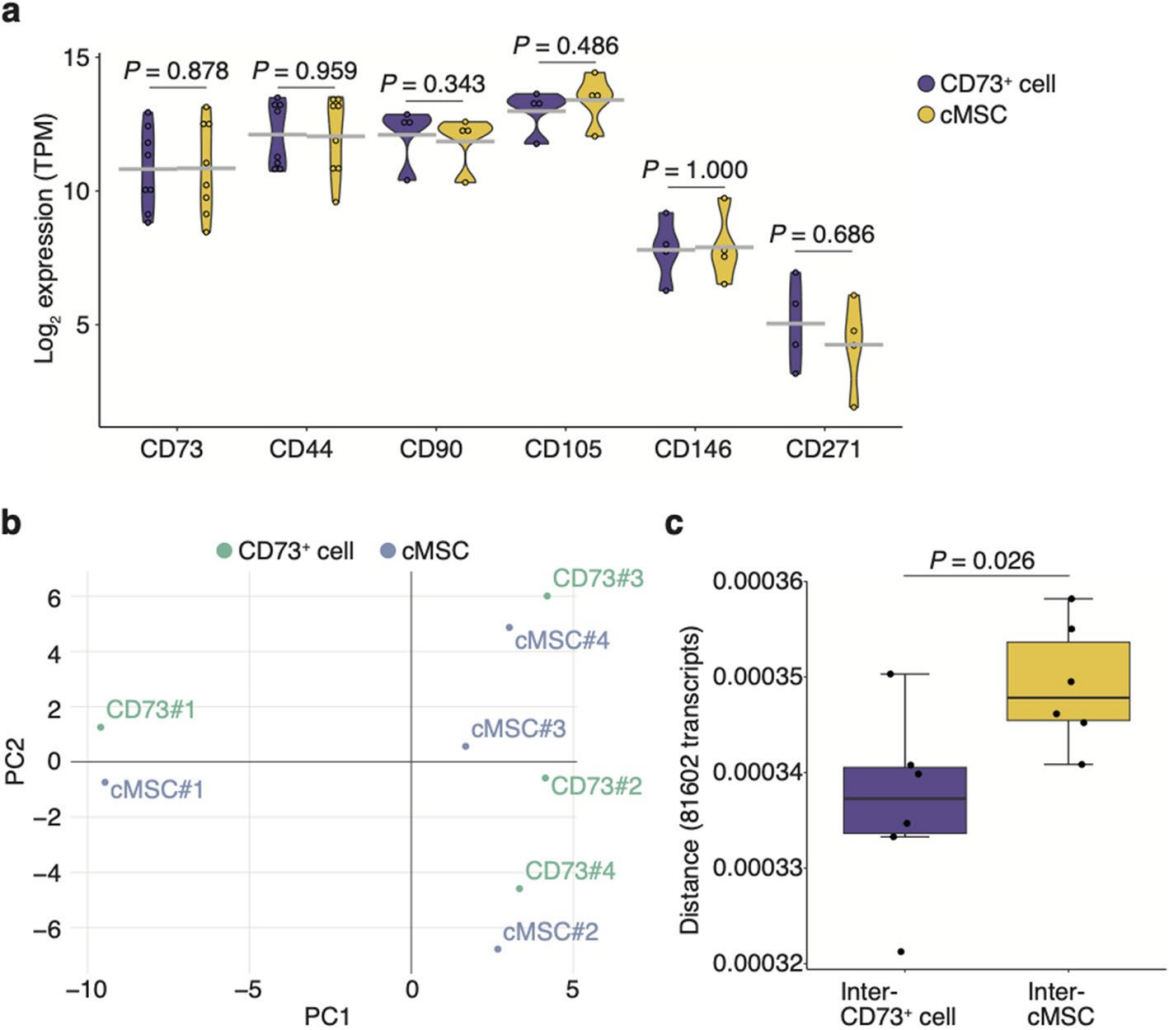
Characterization of homogenous CD73^+^ cell populations and conventional heterogeneous adherent mesenchymal stem/stromal cells (cMSCs).** a** Gene expression (log2) of representative MSC markers in human adipose tissue-derived CD73^+^ cells and cMSCs based on mRNA-seq analysis (*n* = 4). Statistical significance was determined using the Wilcoxon rank-sum test (*P* < 0.05). **b** Principal component analysis showing the relatedness of each sample based on the expression of the top 100 genes (*n* = 4 specimens). **c** Graph showing the interindividual Euclidian distance based on the mRNA expression (log2) of 81,602 transcripts between CD73.^+^ cell populations and cMSCs (*n* = 4 specimens). The cell culture period is approximately 14 days (less than two passages). Statistical significance was determined using the Wilcoxon rank-sum test (*P* < 0.05)

Although the efficacy of CD73^+^ cells and CD73^+^ spheroids in treating inflammatory diseases in these animal models is promising, whether the CD73^+^ cell population is homogeneous remains unknown. Therefore, we reanalyzed the gene expression profiles based on transcription analysis of CD73^+^ cells and cMSCs. No significant differences in the expression of MSC markers, including CD73, CD44, CD90, CD105, CD146, and CD271, were observed between the CD73^+^ cell populations and cMSCs (Fig. 2a). Principal component analysis showed that one donor (donor #1) was distant from the others (Fig. 2b). We compared the distance based on their gene expression between CD73^+^ cells and cMSCs. Notably, the distance between inter-CD73^+^ cells was significantly smaller than that between cMSCs (*P* = 0.026; Fig. 2c). These results indicate that the CD73^+^ cell population has less donor-to-donor variability than heterogeneously adherent MSCs, which makes them clinically applicable.

## Conclusions

In clinical trials of MSCs, the optimal transplantation method for target diseases must be defined; thus, the original tissue, isolation, form, and delivery route of MSCs must be adapted according to the disease. 3D culture–derived MSC constructs may be suitable for treating surgically accessible bone and cartilage defects or IBDs, especially perianal fistulizing CD (Fig. 1). In addition, it is desirable to use MSCs isolated with specific cell-surface markers to avoid interindividual deviation. However, several challenges persist, such as the development of clinical antibodies for isolation and residual antibodies in transplanted MSCs; furthermore, their efficacy and safety need to be considered. Nevertheless, the properties of MSCs render them promising in regenerative medicine.

## Data Availability

The mRNA-seq data included those described in previous studies and were reanalyzed. The mRNA-seq data supporting the findings of the present study were deposited in the Gene Expression Omnibus SuperSeries dataset GSE211637 and GSE265844.

## Abbreviations

AD-MSCs: Adipose tissue-derived MSCs
5-ASA: 5-Aminosalicylic acid
BM-MSCs: Bone marrow-derived MSCs
CCL: C–C motif chemokine ligand
CD: Crohn’s disease
CDAI: Crohn’s disease activity index score
CRP: C-reactive protein
ECM: Extracellular matrix
FACS: Fluorescence-activated cell sorting
FGF: Fibroblast growth factor
IBDs: Inflammatory bowel diseases
IL: Interleukin
MSCs: Mesenchymal stem/stromal cells
TCC-GUI: Tag-Count Comparison Graphical User Interface
Th: T helper
3D: Three-dimensional
TNF-α: Tumor necrosis factor-alpha
TSG-6: TNF-α-stimulated gene 6
UC: Ulcerative colitis
UC-MSCs: Umbilical cord-derived MSCs
